# Effects of a Delphi consensus acupuncture treatment protocol on the levels of stress and vascular tone in women undergoing in-vitro fertilization: a randomized clinical trial protocol

**DOI:** 10.1186/s12906-017-1693-4

**Published:** 2017-04-04

**Authors:** Yan Zhang, Jennifer Phy, Chris Scott-Johnson, Sheila Garos, Jennie Orlando, Samuel Prien, Jaou-Chen Huang

**Affiliations:** 1 Department of Family and Community Medicine, Texas Tech University Health Sciences Center, 3601 4th Street, Lubbock, TX 79430-8143 USA; 2 Department of OB-GYN, Center for Fertility and Reproductive Surgery, Texas Tech University Health Sciences Center, 808 Joliet, Suite 230, Lubbock, TX 79415 USA; 3 Clinical Research Institute (CRI), Texas Tech University Health Sciences Center, 3601 4th Street, STOP 8183, Lubbock, TX 79430 USA; 4 Department of Psychological Sciences, Texas Tech University, 1824 18th Street, MS 42051, Lubbock, TX 79409-2051 USA

**Keywords:** Acupuncture, Delphi consensus protocol, IVF

## Abstract

**Background:**

The variability of published acupuncture protocols for patients undergoing In-Vitro Fertilization (IVF) complicates the interpretation of data and hinders our understanding of acupuncture’s impact. In 2012, an acupuncture treatment protocol developed by a Delphi consensus process was published to describe the parameters of best practice acupuncture for Assisted Reproductive Technology and future research. However, there has been no clinical trial utilizing this protocol to assess the effects of acupuncture. This study aims to ﻿a﻿sse﻿ss the implementation of Dephi consensus acupuncture protocol and to examine the impact of acupuncture on stress and uterine and ovarian blood flow among women between ages 21-42 years seeking IVF.

**Methods/Design:**

This study is a one site prospective, two-arm randomized controlled non-blind clinical trial conducted in a medical school-affiliated fertility center . Participants will be randomized 1:1 into either the acupuncture group or the standard of care (no acupuncture) group using computer generated tables.

Both groups will have 3 regular clinical visits as their standard IVF care during an approximately 2 to 3 weeks window. Women who are randomized into the acupuncture group would receive three sessions based on the Delphi consensus acupuncture protocol in addition to the standard care. The first treatment will be administered between days 6 to 8 of the stimulated IVF cycle. The second session will be performed on the day of embryo transfer at least 1 h prior to the transfer. The third session will be performed within 48 h post-embryo transfer. Participants will be followed for their pregnancy test and pregnancy outcome when applicable.

The outcomes stress and blood flow will be measured by a validated perceived stress scale and vasoactive molecules, respectively.

**Discussion:**

Although recruitment and scheduling could be challenging at times, the Delphi consensus acupuncture protocol was implemented as planned and well-accepted by the patients. Because of the time-specified sessions around patients’ IVF cycle, it is highly recommended to have on-site study acupuncturist(s) to accommodate the schedule.

**Trial Registration:**

ClinicalTrials NCT02591186 registered on October 7, 2015.

**Electronic supplementary material:**

The online version of this article (doi:10.1186/s12906-017-1693-4) contains supplementary material, which is available to authorized users.

## Background

Over the past decades, female subfertility has become a growing problem due to various reasons [1]. According to CDC’s 2011–2013 report, approximately 7.5 million American women aged 15–44 years have impaired ability to conceive or carry a baby to term. The majority of them (6.9 million) seek fertility treatment [2]. By far, In-Vitro Fertilization (IVF) is the most effective treatment as it brings the oocytes and the sperm together and provides an environment for early embryo development, which overcomes barriers such as tubal occlusion, pelvic endometriosis and adhesions [3]. Despite advancements in the embryo culture media, incubation systems and embryo transfer techniques, successful implantation of the embryos is not always guaranteed. Therefore, women often seek complementary treatments, hoping to enhance their reproductive potential and maximize their chance of success [4].

Among the various complementary medicines, acupuncture has gained popularity in patients with subfertility or infertility. However, its benefit in IVF success remains controversial: some reviews suggested a positive effect [5–7], while others argued no proven benefit [8]. Despite the conflicting reports, the majority of the studies agreed that acupuncture is beneficial in reducing stress and enhancing the general wellbeing in women undergoing IVF, but more studies are needed [5, 9, 10].

It is not uncommon that women undergoing IVF are subject to increased stress as both the wait for the outcome and the uncertainty of success cause stress and anxiety [11]. Acupuncture may be beneficial in alleviating the anxiety and the stress, which may adversely affect the outcome through mind and body interaction [12]. It has been proposed that acupuncture makes women undergoing IVF feel more relaxed and optimistic, which might be another mechanism to improve clinical outcomes [13]. While it is not entirely clear how acupuncture works, the literature suggests that acupuncture may work 1) through mediating the release of neurotransmitters which in turns help improve ovulation, regulate the menstrual cycle and reduce stress [4, 5, 14], 2) by altering circulation, which improves uterine and ovarian blood flow [4, 15], and 3) by optimizing immune function [5, 14]. Of all the aforementioned, enhancing blood flow to the uterus and ovaries is physiologically plausible, especially in women undergoing IVF, as enhanced perfusion helps the development of oocytes and the timely maturation of the uterine lining in preparation for embryo implantation. Thus, we identify stress and blood flow as the main outcomes in our study. The results of our study will help us better understand the mechanism by which acupuncture enhances the success of fertility treatment.

To obtain measurable outcomes, it is essential to have a replicable acupuncture protocol. Published acupuncture protocols for patients undergoing IVF vary in the points selected, the frequency and duration of treatment, and the mode of stimulation [16]. This variability further complicates the interpretation of data and hinders our understanding. In 2012, an acupuncture treatment protocol was developed by a Delphi consensus process involving 28 English speaking international and national acupuncturists working in the fertility area involving women undergoing Assisted Reproductive Technology (ART) treatment [16]. This protocol describes the parameters of best practice acupuncture for ART, including a protocol suitable for future research. However, to our knowledge, there has been no clinical trial utilizing this protocol to assess the effects of acupuncture.

The aim of this study is to examine the impact of acupuncture on stress and uterine and ovarian blood flow, which will be measured by a validated stress scale and vasoactive molecules, respectively. We hypothesize that the Delph consensus acupuncture protocol will reduce participant’s stress level and improve uterine and ovarian blood flow. The findings of our study will confirm the feasibility of the Delphi protocol in the setting of clinical trial or clinical practice, and shed light on the mechanism of acupuncture in patients receiving fertility treatment.

## Methods

### Study design

This is a single-center prospective, non-blinded, two-arm randomized controlled clinical trial to assess acupuncture’s impact of stress and uterine and ovarian blood flow of women who undergo IVF. Participants will be randomized 1:1 into either the acupuncture group or the standard of care (no acupuncture) group. Each group will have 3 study visits during their IVF cycle. This trial is registered at ClinicalTrials.gov with identifier NCT02591186 on October 7, 2015.

### Ethics approval and consent to participate

Currently we use the study protocol version 1.11 that was approved by TTUHSC IRB on April 4, 2016 (Study # L15–117). The current clinical research consent form1331 was approved by IRB on September 8, 2016 and will expire on August 23, 2017. The consent form information includes descriptions of the study background and procedures as well as discussion of potential benefits and risks (Additional file 1: Appendix A). Participants will be screened for eligibility by the two fertility specialists. If eligible, the study coordinator will distribute the consent form and discuss any questions or concerns regarding the informed consent. Voluntary signature on the form indicates the women being fully consented to participating in the study.

### Study setting

Following approval by the Texas Tech University Health Sciences Center Institutional Review Board, all women receiving IVF treatment from two fertility specialists at the Center for Fertility and Reproductive Surgery of Texas Tech University Health Sciences Center will be invited to participate in the study. This center performs approximately 130 IVF cycles annually. Historically, patients seeking treatment for infertility are motivated to try complementary and alternative treatments. Therefore, we anticipate a study participation rate of 50% or greater, and a goal of 70 participants it projected to be enrolled in the study over 2 years from December 2015 to December 2017. As our study will be a part of the patient’s standard IVF care, we expect high adherence and retention after enrollment.

### Eligibility criteria

Inclusion criteria: Women between ages 21–42 years seeking IVF will be invited to participate, unless their treatment falls at a time where participation cannot be conducted such as during the absence of key study personnel. Participants must be willing to undergo acupuncture and have no contraindications to needle insertion.

Exclusion criteria: Women currently using alternative therapies such as acupressure, herbal supplements and meditation techniques will be excluded. Women with generalized psoriasis, neuropathy or coagulopathies posing increased risk due to needle insertion will also be excluded.

In the event that embryo growth does not progress or implantation is cancelled for any reason, the subject’s participation in this study will end.

### Randomization

Women who signed the inform consents will be randomized to either the acupuncture group or the control group based upon random numbers generated by the biostatistician at Clinical Research Institute using a computer before the beginning of the trial. The random numbers will be placed in consecutively numbered sealed envelopes. The study coordinator will assign her to either group based on random number indicating the allocation. Women who are randomized into the acupuncture group would receive three sessions of acupuncture performed during stimulation day 6 to 8 and around embryo transfer as described in the acupuncture protocol below. Women who are in the control group will continue their standard IVF treatment. The study is not blinded.

### Acupuncture intervention

**Fig. 1:**
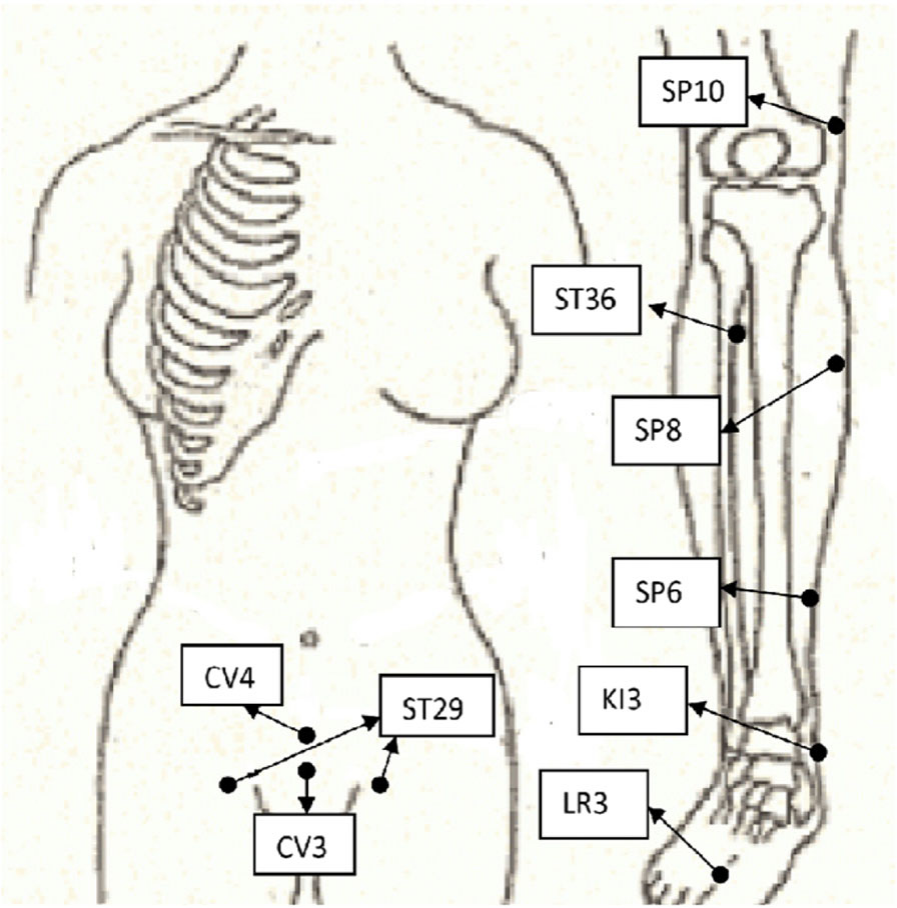
Main body acupoints used in acupuncture protocol

For the intervention group, acupuncture intervention is added in addition to their standard IVF care. An acupuncture treatment protocol is established based on a Delphi consensus protocol developed specifically for patients undergoing IVF [16]. Three semi-standardized sessions of manual acupuncture treatment will be administered during an IVF cycle including embryo transfer (ET). Each session will run about 30 ~ 40 min at the Center for Fertility and Reproductive Surgery. The first treatment includes core points ST29 bilateral, CV4, CV6, SP6 bilateral, SP10 bilateral, and 5 de-stressed points (LI11, LU4, LI4, HT7 and ST36 unilateral) adapted from the clinical set points protocol of Miriam Lee, one of pioneering acupuncturists in the USA [17]. This 13-point session will be administered between days 6 to day 8 of the stimulated IVF cycle. The second session includes points SP8 bilateral, SP10 bilateral, LR3 bilateral, ST29 bilateral, CV4, one selected from HT7/PC6/ EX-HN3 (depending on symptom presentation of women), and auricular acupuncture points Shenmen and Zigong (left side). The 12-point session will be performed on the day of embryo transfer at least 1 h prior to the transfer. Needling sensation, *De qi*, is a composite of unique sensations interpreted as the flow of Qi or ‘the arrival of vital energy’ and will be elicited during the first and second acupuncture sessions. Eliciting methods include local point rubbing before the needle insertion and/or needle rotation after needle insertion. Either patient’s description of *suan* (aching or soreness), *ma* (numbness or tingling), *zhang* (fullness, distention, or pressure), and *zhong* (heaviness) or the acupuncturist’s feeling of needle grasping as tense, tight, and full at the needling region will be considered as *De qi* [18]. No other stimulation will be elicited during needle retention. The third session includes GV20, KI3 bilateral, ST36 bilateral, SP6 bilateral, PC6 bilateral and auricular acupuncture points Shenmen and Zigong (right side). The 11-point session will be performed within 48 h post-embryo transfer. Main points are shown in Fig. 1 and all points will be located based on WHO Standard Acupuncture Point Locations 2008 version [19]. Disposable stainless-steel acupuncture needles (0.16 × 15 mm, 0.18 × 30 mm and 0.20 × 40 mm, DBC Spring Ten, Korea) will be used. Needles will be inserted manually to a depth varying from 10 ± 5 mm to 25 ± 5 mm at the acupoints depending on the location and patient’s physical figure. Needle retention time will be 25 min. A laminated acupuncture protocol describing the timing and acupoints for each session is stored with all acupuncture equipment so the acupuncturist can check and ensure consistency and accuracy during the treatment.

### Comparison

Standard IVF care at the center will be used to compare with acupuncture intervention in addition to the standard IVF care. Women who are randomly assigned in the control group will continue standard IVF treatment and will be offered three vouchers for acupuncture treatment to be used after their IVF cycle for any conditions they choose to treat. These vouchers will be provided after the participant’s embryo transfer and may be used within 1 year from the date of the embryo transfer (e.g., control group embryo transfer on 6/1/2015, 3 acupuncture vouchers valid from 6/1/2016 through 6/1/2017). The delayed use of the acupuncture vouchers is intended to avoid use of the acupuncture sessions during pregnancy in the control group which could potentially affect outcome data comparisons between the two groups. If a subject leaves the study early, she will receive the number of acupuncture vouchers equal to the number of visits completed.

### Outcome measures

#### Stress

To determine whether acupuncture reduces stress, study participants are asked to complete a validated Perceived Stress Scale (PSS) to assess self-perceived stress. PSS is the most widely used psychological instrument for the measurement of perceived stress. It has reported Cronbach’s α between .84 and .86 and the test-retest reliability of .85 [20]. It is a measure of the extent to which situations in one’s life are appraised as stressful [20]. The participants rate their feelings and thoughts during THE PAST MONTH. In each of the 10 questions, they will be asked HOW OFTEN they felt or thought a certain way from never (0), almost never (1), sometimes (2), fairly often (3), to very often (4). Item ratings will be summed, with higher scores indicating more perceived stress.

#### Blood flow

The blood perfusion to organs is determined by local vascular tones, which in turn is controlled by the balance of two key vasoactive molecules, prostacyclin and thromboxane. The former relaxes the vascular smooth muscle, dilates blood vessels, and prevents platelet aggregation; the latter contracts the vascular smooth muscle, constricts blood vessels, and promotes platelets aggregation [21]. Both thromboxane and prostacyclin have short half-lives up to an hour, but their urinary metabolites, thromboxane B_2_ (TBX_2_), and 2, 3-dinor-TXB_2_ (both from thromboxane), and 6-keto-prostaglandin F_1 α_ (6-keto PGF_1α_), and 2, 3-dinor-6-keto-PGF_1α_ (both from prostacyclin) are stable [22]. If the concentrations of these metabolites are within expected values, it should be fairly easily determined using commercially available EIA assay kits. Therefore, we plan to collect the urine samples to measure stable metabolites of thromboxane and prostacyclin, 2, 3-dinor-TXB_2_ and 2, 3-dinor-6-keto-PGF_1α_, respectively.

### Study visit and data collections

After randomization, each participant will have three study visits during their IVF cycle within an approximately 2 to 3 weeks window. The table below depicts the timeline of study activities (Table 1).

**Table 1:** The schedule of enrollment, interventions, and assessments

	Study Period					
	Enrollment	Allocation	Post-allocation			Close-out
Timepoint	*SE* *(ongoing)*	V_0_ (ongoing)	*V*_*1*_	*V*_*2*_	*V*_*3*_	*M*_*12–24*_
Enrolment:						
Eligibility screen	X					
Informed consent	X					
Allocation		X				
Interventions:						
*Acupuncture*			I	I	I	
*Standard IVF Care*			C + I	C + I	C + I	
Assessments:						
*Demographics and Study information*	X	X	X	X	X	X
*PSS* ^a,b^			X	X	X	
*Urine Collection* ^c,d^			X	X	X	

*SE* Study Entry

*V0* Routine IVF visit before day 6 stimulation, *V1* Cycle days 6–8 of IVF stimulation, *V2* Day of embryo transfer and before transfer, *V*_*3*:_Within 48 h Post-embryo transfer, *M*_*12-24*_Study m﻿onths 12 to 24

^a:^ PSS will be collected prior to each acupuncture session for intervention group

^b:^PSS will be collected prior to each monitoring session for control group

^c:^Urine sample will be taken prior to and after each acupuncture session for intervention group

^d:^Urine sample will be taken prior to and after the monitoring visit for control group

PSS and sterile wide-mouth urine containers will be pre-labeled and made available at the clinic prior to the visit. The PSS will be distributed to the participants and collected by a clinical nurse. In the event that the embryo transfer must be postponed for clinical reasons, the post-treatment questionnaire will be performed 3 to 7 days after the oocyte retrieval. The participants will be asked to collect approximately 30 ml mid-stream urine in the container. The clinical nurse will collect the urine samples and store them with de-identified labels in the clinical refrigerator. The study coordinator will pick up the PSS and urine sample. All information on the PSS will be entered in the study database using Microsoft Excel. Urine samples are taken to the Texas Tech University Health Sciences Center OB-GYN laboratory. Prior to first analysis, all urine samples will be prepared by centrifugation, then frozen and held at -70 °C. All samples will be assayed simultaneously using ELISA kits to reduce inter-assay errors.

Other information to be collected includes demographics such as age and race etc., and study information such as acupuncture date/time or visit date/time, urine collection date/time, pregnancy test results, and pregnancy outcome. In the case of discontinuing of the study or the IVF treatment, reasons of exit will be documented. Each participant will be assigned a unique identification number at the time of enrollment, and all data will be de-identified and labeled with the subjects’ ID number then entered in the study database using Microsoft Excel. All de-identified data will be stored securely on encrypted devices accessible only to study investigators in accordance with TTUHSC policies.

#### Safety and adverse event report

Participation in this study will not incur additional risks related to *IVF* itself. Those who are randomized to receive acupuncture will undergo acupuncture in addition to their standard care. Acupuncture is generally considered safe when performed by a trained professional [23]. The risks of acupuncture may include occasional dizziness, bleeding, bruising, and potential anxiety. Women receiving IVF treatment are required to administer medication via subcutaneous and intramuscular injections. It is estimated that they would have had 80–100 injections before the first acupuncture. Compared with the larger, hollow syringe needles used to deliver IVF medicine, discomfort caused by the smaller, solid acupuncture needles will be similar but at a much reduced level. A form is used to document the date/time of each session as well as side-effects or discomfort during the session. In case of adverse event, it will be documented and reported to the primary investigator, who, based on the severity of the condition, will take appropriate action(s). Due to the small scale of the study, a data monitoring committee (DMC) is not needed. A mid-term interim analysis is planned to be done by the study statistician assessing the outcomes. The primary investigator will have access to these interim results and make the final decision to continue or terminate the study earlier than planned. This study is also under institutional routine auditing to ensure study quality and conduct.

#### Statistical analysis plan

Study power consideration: With 35 patients allocated to each group, assuming the Student’s *t*-test will be used to determine the differences between the acupuncture and control groups, effect sizes d > 0.6 will be found statistically significant with alpha .05 and power .80. In practical terms, since the Perceived Stress Scale (PSS) validation samples showed a mean score about 13.5 with variance = 6.2, only averaged differences greater than 1.5 raw score points on PSS scores will be deemed statistically significant. We will use the data obtained from this study to perform power analysis to help estimate the adequate sample size needed for future studies.

Descriptive statistics and bivariate tests (e.g., correlation, t-test, and Chi-square test) will be utilized to summarize sample demographics and examine distributional properties and relations of the study outcomes. Then, general/generalized linear mixed modeling (GLMM) analysis will be conducted to assess effect of the acupuncture on each outcome. To be specific, we will estimate overall difference in stress level and blood flow between the acupuncture and control groups (group effect), change during the study period (time effect), and group difference in this change (group-by-time interaction). A significant group effect or group-by-time interaction will indicate a significant impact of the intervention.

## Discussion

Since starting the enrollment in December 2015 after IRB approval, we have enrolled 31 patients total, 16 in the acupuncture group and 15 in the control group in the first 12 months, slightly less than expected 35 enrollees in 1 year. Over-strict inclusion criteria and timing and were the main barriers of slower enrollment (about one enrollment per month) at the first 4 months of the study. Originally, we had one criterion to exclude “women with a history of previous acupuncture experience”. During the early stage of recruiting, we found more than expected women had acupuncture experiences not necessary related to fertility, the aforementioned criterion excluded those women otherwise would be eligible. After discussion, the study team agreed to remove this exclusion criterion and collect patient’s history of prior acupuncture experience as a variable in the database starting in March 2016. We have observed substantial increase of enrollment since April 2016. The holiday season after Thanksgiving to New Year appeared not ideal for recruitment and enrollment. On the one hand, the Center for Fertility and Reproductive Surgery closes routinely in December for annual lab calibration. During December 2016 and January 2017 specially, IVF cases were not performed due to building construction and center relocation. Although temporarily, this limited our ability to recruit the patients. On the other hand, the study team members taking different length of vacation leave in addition to the institutional holidays during this time may have slowed the enrollment process as well. Regular or ever better enrollment pace is expected after January 2017 as the Center has higher patient capacity after r﻿elocation. In addition to timing, it is worth noting that distance from patient’s home to the clinic poses another barrier during recruitment. Although we make effort to accommodate patient’s IVF cycle, patients residing hours away from the center tended to decline to participate due to the travel. Carefully taking the eligibility criteria, clinical scheduling and patient’s physical location into consideration may help make a more effective recruitment and enrollment planning in the future.

In addition to the issues we encountered during enrollment, streamlining the schedule between patient’s usual physician/IVF visit and acupuncture session has been challenging. Due to credentialing regulations of the hospital, we only have one credentialed acupuncturist who can provide on-site treatment in the Center for Fertility and Reproductive Surgery for the study patients. It is important to point out that the on-site acupuncture session is well received by patients because there is no need to make extra appointments with the acupuncturist and it saves their trip going back and forth to an off-site clinic. Every effort has been made to accommodate patient’s IVF cycle. Although we have not lost any patient enrolled in the acupuncture group, we were not able to enroll patients when the sole acupuncturist was not available. Therefore, a substitute acupuncturist is recommended for future studies.

In spite of the difficulties mentioned above, the study protocol went well in general. It is feasible to conduct a Delphi consensus acupuncture protocol in the setting of clinical trial or clinical practice. The effect of such protocol will be assessed after we complete the study.

## Additional file

Additional file 1: Appendix A. Study Inform Consent. (PDF 97 kb)
