# Supplementary material for: Effects of a Delphi consensus acupuncture treatment protocol on the levels of stress and vascular tone in women undergoing in-vitro fertilization: a randomized clinical trial protocol
Source: BMC Complement Altern Med. 2017 Apr 4;17:197. doi: 10.1186/s12906-017-1693-4 (PMC5381072; doi:10.1186/s12906-017-1693-4)
Supplement: Additional file 1: — Appendix A. Study Inform Consent. (PDF 97 kb) [file 12906_2017_1693_MOESM1_ESM.pdf]

This consent form is not valid without a TTUHSC IRB stamp in the lower left corner of each page.

CONSENT TO TAKE PART IN A RESEARCH STUDY

This is a research study for people who voluntarily choose to take part. Please take your time to make a decision, and discuss the study with your personal doctor, family and friends if you wish.

STUDY TITLE: Effect of Acupuncture on Vascular Biomarkers and Psychological Well-Being of Women Undergoing In Vitro Fertilization (IVF)

INVESTIGATORS: Jennifer Phy, DO
Jaou-Chen Huang, MD
Yan Zhang, PhD
Samuel Prien, PhD
Sheila Garos, PhD
Jennie Orlando, MD

CONTACT TELEPHONE NUMBERS:

Dr. Phy: 806-743-4256

Clinical Research Institute: 806-543-8994

(You may contact the investigators at the numbers listed above at any time if you develop any of the conditions listed in Question #6 of this form or if you have any unexpected complications.)

INSTITUTION: Texas Tech University Health Sciences Center, Lubbock, Texas

1. Why is this study being done?

Treatment for infertility is often expensive and time-consuming. It is also stressful for the patient and her family. Stress has been shown to negatively affect the success of fertility treatment. Acupuncture has been found to reduce stress. The purpose of this study is to find out how acupuncture affects the stress levels and psychological well-being of women undergoing IVF.

Acupuncture is a type of complementary and alternative medicine that originated in China 5,000 years ago. It is based on a theory that energy and blood circulate through a system of channels that connect internal organs with external organs and tissues. By stimulating certain points of the body surface where the channels reach (acupoints), the flow of energy and blood can be regulated. The acupuncture specialist will use needles to stimulate the points on the body.

2. How many people will take part in this study?

About 70 women will take part in this study.

3. **Why am I being asked to take part in this research study?**
You are being asked to take part in this study because you are seeking fertility treatment at the Center for Fertility & Reproductive Surgery.

4. **What will happen during this study? What will be done that is different from my usual care?**
After consent is given, the following will occur:
    - You will be randomly assigned (like flipping a coin) to either the study group (will receive acupuncture) or the control group (no acupuncture).
    - All participants will be asked to attend three study visits.
        - Visit 1 will take place between days 6 and 8 of the IVF cycle.
        - Visit 2 will take place on the day of embryo transfer.
        - Visit 3 will take place after embryo transfer but prior to taking a pregnancy test. This visit will take place between 24-48 hours after embryo transfer, if possible. Please let the study team know if you can make it to this visit during this timeframe.
    - We will collect information from your medical record such as your age, race, and pregnancy test results. If you become pregnant, we will collect information about the pregnancy outcome after your due date.
    - You must agree not to take NSAIDs (Advil, Ibuprofen, etc…) during your IVF Treatment.

    <u>**Experimental Group (Acupuncture Group)**</u>
    - You will receive your standard IVF care at the Center for Fertility and Reproductive Surgery.
    - At each of 3 study visits, you will also receive a standard acupuncture treatment protocol that is specifically made for patients having IVF.
    - You will be given an informational sheet containing detailed information on the acupuncture procedure.
    - Needles will be inserted manually at the acupoints that are believed to be effective for patients having IVF. These acupoints are located in the ear, abdomen and pelvic areas, and on the lower leg and foot. At each visit, 5-9 acupoints will be used. On the day of embryo transfer, only acupoints on the ear and below the belly button will be used. Needles will be inserted less than 1 inch, depending on the location and your physical figure.
    - Up to 20 disposable needles per session may be used. The length of these needles will range from 1.5cm-4cm (shorter than 2 inches). The needle size used will depend on the location and your physical figure.
    - You may feel a small prick as the needle passes through the skin. However, the needles are as fine as a human hair. Many patients do not feel any pain at all.
    - Each acupuncture session will take place at either the Family Medicine Clinic or Center for Fertility and Reproductive Surgery. Treatments will last about 30-40 minutes.
    - You will give a urine sample before and after each acupuncture session.
    - You will also complete a questionnaire about your stress level before each acupuncture session.

Control Group (Standard Care Group)

- You will continue your usual IVF care at the Center for Fertility and Reproductive Surgery.
- At each of 3 study visits, you will be asked to come to the Center for Fertility and Reproductive Surgery and sit for 30-40 minutes. This visit will take place between 24-48 hours after embryo transfer, if possible. Please let the study team know if you can make it to this visit during this timeframe.
- You will give a urine sample at the beginning and end of each visit.
- You will complete a stress questionnaire at each study visit.

Poor Response to Egg Retrieval:

- If you have a poor response to initial medication doses you may continue to be in the study if you plan on having retrieval at a later date.
- You will **NOT** be randomized again, you will be in the same group as you previously were assigned.
- If this is the case you will proceed with study visits 1 – 3.

5. How much of my time will this study take? How long will I be in the study?

You will be asked to attend three study visits that will each take about 30-40 minutes of your time during your 8-week IVF cycle. Information about you will be kept until completion of the study, or for about 3 years.

6. What are the risks and/or discomforts to me if I join this study?

Risks related to acupuncture (study group)

- Dizziness from fear of needles
- Minor bleeding after removal of the needles
- Minor bruising after removal of the needles
- Potential anxiety
- During acupuncture, you may feel a sensation of heaviness, numbness, a slight dull ache or a twitch which usually indicates that the vital energy has arrived at the acupoint. Very rarely will you feel sharp pain. If you do, inform your acupuncturist so that an adjustment can be made.

Other risks related to the study (both groups)

- Possible loss of confidentiality

7. Will there be any added risks to me from this study if I am a female?

No.

8. Are there any benefits to me if I take part in this study?

It is possible that taking part in this study will not benefit you.

9. What other choices do I have if I do not take part in the research study?

Taking part in this study is voluntary. If you do decide not to take part in this study, you will continue on your regular IVF course. You will not receive any additional intervention, and no data will be collected for this study.

10. **What about confidentiality and the privacy of my records?**
We will keep your involvement in this research study confidential to the extent permitted by law. In addition to the staff carrying out this study, others may learn that you are in the study. This might include federal regulatory agencies such as the Food and Drug Administration (FDA) and the Office for Human Research Protection (OHRP), Texas Tech University Health Sciences Center (TTUHSC) representatives, and the TTUHSC Institutional Review Board (a committee that reviews and approves research). These people may review and copy records involving this research. A copy of this document may be placed in your medical record.

Study results that are used in publications or presentations will not use your name.

11. **Who is funding this study?**
The Laura W. Bush Institute for Women's Health and University Medical Center Women's Health Research Scholar Fund are providing the space and supplies for this study. No one on the research staff will receive anything of value from other agencies, organizations, or companies to carry out this research.

12. **Will it cost me anything to take part in this research study?**
No. All study procedures will be paid for by the study.

13. **Will I receive anything for taking part in this research study?**
All study participants will receive 3 acupuncture treatment sessions at no cost to them.

Participants in the study group will receive the 3 treatment sessions during the course of the study, as described above. Participants in the control group will receive 3 vouchers for acupuncture sessions at a later date. If you are in the control group, you will be given the 3 vouchers after your final study visit. You may redeem the vouchers starting 1 year after the date of embryo transfer. The voucher will be good for 1 year from that date. *(Example: you're your embryo transfer takes place on 6-1-2015, your vouchers will be good 6-1-2016 through 6-1-2017.)*

14. **Does anyone on the research staff have a personal financial interest in this study?**
No.

15. **What if I am hurt by participating in this study?**
Texas Tech University Health Sciences Center and its affiliates do not offer to pay for or cover the cost of medical treatment for research related illness or injury. No funds have been set aside to pay or reimburse you in the event of such injury or illness unless specifically stated.

If you have a research related illness or injury, care will be available to you as usual, but you and/or your medical or hospital insurance company will be responsible for the cost of treatment. Before entering this study, you should check whether your insurance company might limit your insurance coverage if you take part in a research study.

16. **What are my rights as a voluntary participant?**
    - Taking part in this study is your choice. You may choose not to be in it. If you decide not to be in the study, it will not affect any medical care, benefits or rights to which you are entitled.
    - If you sign this form, it means that you choose to be in the study. If new information becomes available during the study that may affect your willingness to take part in the study, you will be told.

17. **Can I stop being in the study?**
    - You may leave the study at any time. If you do, discuss it with the investigator, who will help you leave the study in the safest way.
    - If you leave the study, your right to standard medical care will continue.
    - If you leave the study, we cannot remove any information we have collected to that point.
    - If you are in the control group and leave the study before your final visit, you will still receive one acupuncture voucher for each visit completed.

18. **Can someone else end my participation in the study?**
    Under certain circumstances, the investigators, TTUHSC, or the study sponsor may decide to end your participation in this research study earlier than planned. This might happen if your embryo implantation is cancelled for any reason.

19. **What if I have questions?**
    For questions about this study, contact the Investigator, Dr. Jennifer Phy, at 806-743-4256.

    If you would like to speak to someone who is not involved in the study about your rights as a participant, research-related injuries, or any other matter related to the study, you can call the TTUHSC EthicsPoint Hotline: 1-866-294-9352.

    Or, you can file an EthicsPoint report online: https://secure.ethicspoint.com/domain/media/en/gui/12958/index.html. Please choose the “Regulatory Compliance” option when making an online report.

    A description of this clinical trial will be available on <u>www.ClinicalTrials.gov</u>, as required by U.S. Law. This Web site will not include information that can identify you. At most the Web site will include a summary of the results. You can search this Web site at any time.

Your signature indicates that

- **this research study has been explained to you;**
- **you've been given the opportunity to ask questions;**
- **you accept your responsibility to follow the instructions given to you by the research team regarding study participation and, if applicable, research medication;**
- **you agree to take part in this study.**

You will be given a signed copy of this form.

____
Printed Name of Subject

____ ______________________
Signature of Subject Date Time

____ ______________________
Signature of Parent/Guardian Date Time
or Authorized Representative

(Investigator: Witness is to sign below ONLY if subject is unable to read the written consent form. If they or authorized representative can read the consent form, leave this section BLANK)

_____ Subject was unable to read and understand the written consent.

The elements of informed consent required by 45 CFR 46.116 and 21 CFR 50 have been presented orally to the subject or the subject's authorized representative in a language understandable to the subject or representative.

____ ______________________
Signature of Witness to Oral Presentation Date Time

(Investigator: The following is to be signed for ALL consent forms)

I have discussed this research study with the subject and his or her authorized representative, using language that is understandable and appropriate. I believe I have fully informed the subject of the possible risks and benefits, and I believe the subject understands this explanation. I have given a copy of this form to the subject.

____ ______________________
Signature of authorized research personnel who Date Time
conducted the informed consent discussion

TEXAS TECH UNIVERSITY HEALTH SCIENCES CENTER (TTUHSC)

AUTHORIZATION TO USE AND/OR DISCLOSE YOUR PROTECTED HEALTH INFORMATION for a RESEARCH STUDY

STUDY TITLE: Effect of Acupuncture on Vascular Biomarkers and Psychological Well-Being of Women Undergoing In Vitro Fertilization (IVF)

This form is intended to tell you about the use and/or disclosure (sharing) of your personal **Protected Health Information** (PHI) if you decide to participate in the research study described on the previous pages. The health information about you that may be used or disclosed is described below. This information is usually found in your medical records. Only the health information about you that is needed for this research study will be used or disclosed. When you consider taking part in this research study, you are also being asked to give your permission for your Protected Health Information to be released from your doctors, clinics, and hospitals to the research personnel approved for this research study. This Authorization specifically relates to the research study described in the attached Informed Consent document.

1. This Authorization is valid indefinitely or until such time as legal requirements will allow this Authorization to be destroyed.

2. If you choose to cancel this Authorization, please give notice in writing to:
   Institutional Privacy Officer
   Office of Institutional Compliance
   3601 4th St MS 8165
   Lubbock TX 79430

If you sign this Authorization, the following persons, groups or organizations may rely on this Authorization to disclose your Protected Health Information to the Principal Investigator and other research personnel who are conducting this Study:

- your treating physicians and healthcare providers and their staff,
- associated healthcare institutions and hospitals where you have or may receive care.

While this research study is in progress, the Principal Investigator or research personnel working on this study will inform you whether or not you will be allowed to see the research related health information that is created about you or collected by the research personnel prior to the end of the study. After the study is finished you may request this information as allowed by the TTUHSC Notice of Privacy Practices.

The Protected Health Information that you authorize to be used or disclosed for research purposes may include your current or future health information from some or all of your health records, including:

• hospital records and reports	• immunizations
• admission history, and physical examination	• allergy reports
• X-ray films and reports; operative reports	• prescriptions

• laboratory reports, treatment and test results (including sexually transmitted diseases, HIV or AIDS)<br>• any other Protected Health Information needed by the research personnel listed above.<br><br>(*use separate form for disclosure of psychotherapy notes)	• consultations<br>• clinic notes<br>• mental health records<br>• alcohol / substance abuse records

For the purposes of this study, your Protected Health Information may need to be reviewed or disclosed to individuals or organizations within and/or outside of TTUHSC who sponsor, approve, assist with, monitor or oversee the conduct of research studies. This includes, but is not limited to, the TTUHSC Institutional Review Board, TTUHSC compliance reviews, the US Food and Drug Administration (FDA) or governmental agencies in other countries. Some of these individuals or organizations may share your health information further, and your health information may not be protected by the same privacy standards that TTUHSC is required to meet.

If you choose to sign this Authorization form, you can change your mind about this later. If you change your mind, send a letter to the person identified above telling us to stop collecting and sharing your Protected Health Information. When we receive your request, you may be asked to leave the research study if all the necessary information has not been collected. We may still use the information about you that we have already collected. We need to know what happens to everyone who starts a research study, not just those people who stay in it.

You have the right to refuse to sign this form. If you choose not to sign this form, your regular health care will not be affected. However, not signing this form will prevent you from participating in this research study and prevent you from receiving research related health care services provided under this study.

I have had the opportunity to review and ask questions regarding this Authorization to use or disclose my personal health information, and I will receive a copy of this form. By signing this Authorization, I am confirming that it reflects my wishes.

____
Printed Name

____________________________________ ____________________
Signature of Individual or Authorized Representative or Authorized Representative — Date — Time

____
If applicable, Relationship of Authorized Representative or Authority to Sign
